# Bibliometric Analysis of Quantitative Electroencephalogram Research in Neuropsychiatric Disorders From 2000 to 2021

**DOI:** 10.3389/fpsyt.2022.830819

**Published:** 2022-05-23

**Authors:** Shun Yao, Jieying Zhu, Shuiyan Li, Ruibin Zhang, Jiubo Zhao, Xueling Yang, You Wang

**Affiliations:** ^1^Department of Psychology, School of Public Health, Southern Medical University, Guangzhou, China; ^2^Department of Neurosurgery, Huashan Hospital, Fudan University, Shanghai, China; ^3^Department of Rehabilitation Medicine, School of Rehabilitation Medicine, Southern Medical University, Guangzhou, China; ^4^Department of Psychiatry, Zhujiang Hospital, Southern Medical University, Guangzhou, China

**Keywords:** bibliometrics, quantitative electroencephalogram, neuropsychiatric disorders, CiteSpace, VOSviewer

## Abstract

**Background:**

With the development of quantitative electroencephalography (QEEG), an increasing number of studies have been published on the clinical use of QEEG in the past two decades, particularly in the diagnosis, treatment, and prognosis of neuropsychiatric disorders. However, to date, the current status and developing trends of this research field have not been systematically analyzed from a macroscopic perspective. The present study aimed to identify the hot spots, knowledge base, and frontiers of QEEG research in neuropsychiatric disorders from 2000 to 2021 through bibliometric analysis.

**Methods:**

QEEG-related publications in the neuropsychiatric field from 2000 to 2021 were retrieved from the Web of Science Core Collection (WOSCC). CiteSpace and VOSviewer software programs, and the online literature analysis platform (bibliometric.com) were employed to perform bibliographic and visualized analysis.

**Results:**

A total of 1,904 publications between 2000 and 2021 were retrieved. The number of QEEG-related publications in neuropsychiatric disorders increased steadily from 2000 to 2021, and research in psychiatric disorders requires more attention in comparison to research in neurological disorders. During the last two decades, QEEG has been mainly applied in neurodegenerative diseases, cerebrovascular diseases, and mental disorders to reveal the pathological mechanisms, assist clinical diagnosis, and promote the selection of effective treatments. The recent hot topics focused on QEEG utilization in neurodegenerative disorders like Alzheimer's and Parkinson's disease, traumatic brain injury and related cerebrovascular diseases, epilepsy and seizure, attention-deficit hyperactivity disorder, and other mental disorders like major depressive disorder and schizophrenia. In addition, studies to cross-validate QEEG biomarkers, develop new biomarkers (e.g., functional connectivity and complexity), and extract compound biomarkers by machine learning were the emerging trends.

**Conclusion:**

The present study integrated bibliometric information on the current status, the knowledge base, and future directions of QEEG studies in neuropsychiatric disorders from a macroscopic perspective. It may provide valuable insights for researchers focusing on the utilization of QEEG in this field.

## Introduction

Electroencephalography (EEG) is a tool for recording spontaneous electrical activity generated in the cerebral cortex using multiple electrodes placed on the scalp (1), providing real-time assessment of cerebral physiological functions (2). Since the German psychiatrist Hans Berger first tried to record human cerebral electrical activity from the scalp in 1928 (3), EEG technology has continued to develop, which is currently one of the most influential noninvasive tools available to clinicians for evaluating a patient's neurophysiological functions (4).

Quantitative EEG (QEEG) techniques separate complex EEG signals into components such as amplitude, frequency, and compress time, permitting the display of several hours of data on one image (5). QEEG empowers a neurologist or a psychiatrist's unprecedented ability to look at summarized EEG information, which was not previously possible with a visual examination of EEG traces. More importantly, QEEG can provide an objective, replicable measure of brain functions that is less dependent on subjective or behavioral reports that may vary across settings and informants (6). Therefore, QEEG can be used in more productive ways than non-QEEG to identify and categorize neuropsychiatric diseases, as well as to predict the outcome of therapeutic intervention (7).

To date, several studies have summarized the development of QEEG methodology (8–10) or systematically reviewed the research papers on the possible use of QEEG as a biomarker in adult or child psychiatric disorders (11, 12) and in Alzheimer's disease (13). However, to our knowledge, there is no existing study to analyze the developing status of the QEEG research field from a macroscopic perspective.

Bibliometrics, an important branch of intelligence science (14), uses the literature system and bibliometric characteristics as the research object and conducts quantitative and qualitative analyses of the literature (15). In recent years, bibliometric analysis has been applied to visualize the knowledge status, features, evolution, and emerging trends in various research fields (16). It can help scholars extract quantitative information on distribution by country/region, institution, author, journal, research hot spots, and frontiers in a particular field in a short time, providing in-depth reviews and insights about the research field (17). Therefore, the present study used bibliometric tools to analyze the QEEG studies in neuropsychiatric disorders from 2000 to 2021 to provide a comprehensive overview.

The specific research questions in the present study for QEEG research in neuropsychiatric disorders were as follows: 1) What are the overall publication trends, the geographic distributions, the most important journals, and who are the potential collaborators? 2) What is the knowledge framework in this field in terms of research hot spots and knowledge base? 3) What are the future directions of this field?

## Method and Data Source

### Data Collection

**Figure 1:**
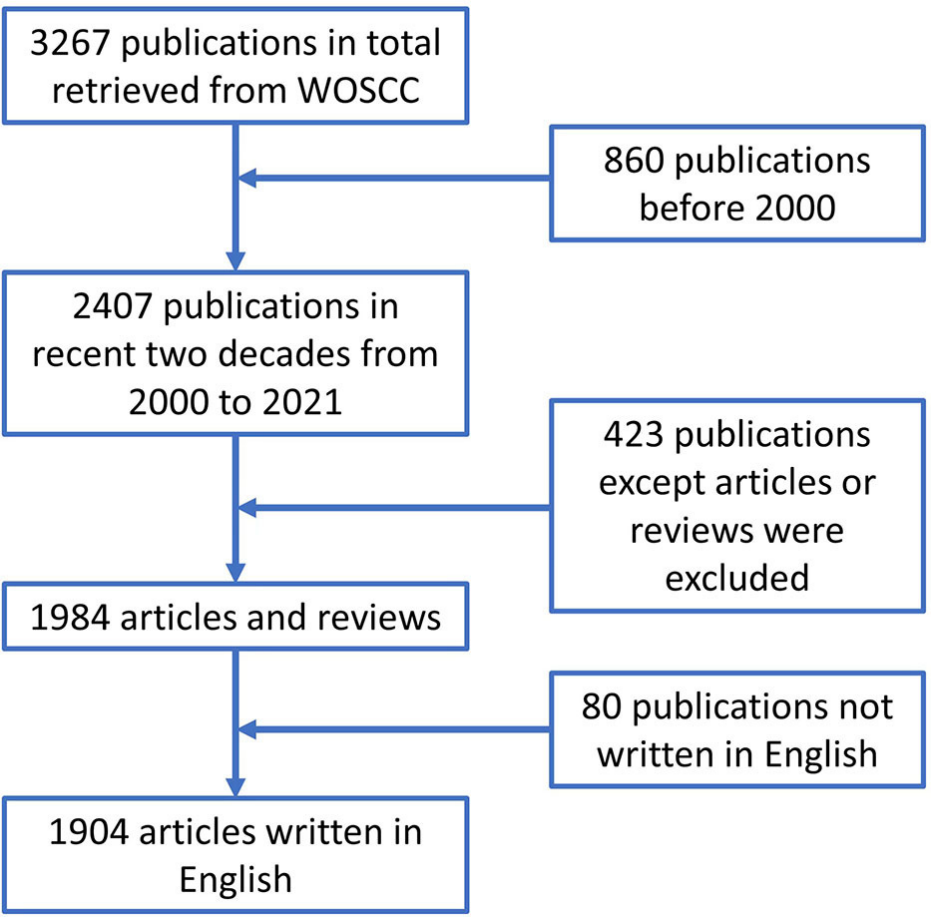
Flow diagram of the inclusion process.

Data for the present study were retrieved from the Web of Science Core Collection (WOSCC) database in January 2022. We used TS = [(quantitative electroencephalography) OR (QEEG) OR (quantitative EEG)] AND WC = [(neurology) OR (psychiatry) OR (neuropsychiatry)] as the search terms, where “TS” represents term subject and “WC” represents “Web of Science categories.” The time limitation was between 1 January 2000 and 31 December 2021. Only literature published in English was included, and duplicated articles were deleted. To avoid bias due to daily database updates, we performed the literature retrieval from WOSCC on a single day, that is, 27 January 2022. A total of 1,904 publications were included and consisted of original articles and reviews. The search strategy is depicted in Figure 1.

### Analysis Tools

All the collected data were converted into TXT format and exported for further visual analysis by bibliometric software, including CiteSpaceV (5.8.R3) and VOSviewer (1.6.16), and the online literature analysis platform (https://bibliometric.com/). Visualized information, such as yearly output, subject categories of WOS, and impact factor (IF), was analyzed based on the function of literature analysis on WOSCC. The number of countries, institutions, and international collaborations were analyzed by bibliometric.com. CiteSpaceV was used to perform collaboration network analysis (including authors, institutions, journals, co-cited journals, co-cited authors, and co-cited references) and related centrality. The specific parameters used in CiteSpaceV were set as follows. For the selection of time slices, a slice of 1 year was used for determining the connection strength, and cosine was used for this purpose. For the threshold, we selected the top 50 nodes in each time slice. Moreover, the pruning used a pathfinder and merged network. VOSviewer was used to analyze the keywords. The keyword co-occurrence map in VOSviewer only includes terms that appear at least 15 times under the binary count. The purpose of the algorithm is to ensure that the terms that occur more frequently have larger bubble images and the terms with high similarity are close to each other with a similar color. Finally, the keyword overlay map was used based on the occurrence of keywords to visualize the emerging topics from 2000 to 2021.

## Results

### Annual Publications

**Figure 2:**
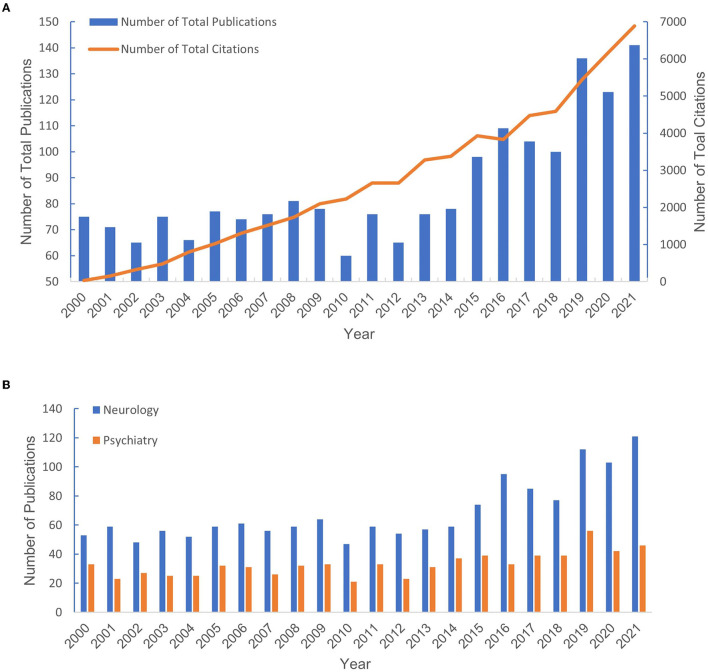
The number of publications from 2000 to 2021. **(A)** The number of publications in neurology and psychiatry. **(B)** The total number of publications and the percentage of total publications.

A total of 1904 publications that met the retrieval criteria were included in further analysis. The total number of annual publications is shown in Figure 2A, which depicts an increasing trend from 2000 to 2021. As shown in Figure 2B, the trend of the annual publications in the neurology field showed steady growth. However, the yearly output of articles in the psychiatry area increased relatively slowly, indicating that the application of QEEG in psychiatric disorders requires more attention.

### Analysis of Countries and Institutions

**Figure 3:**
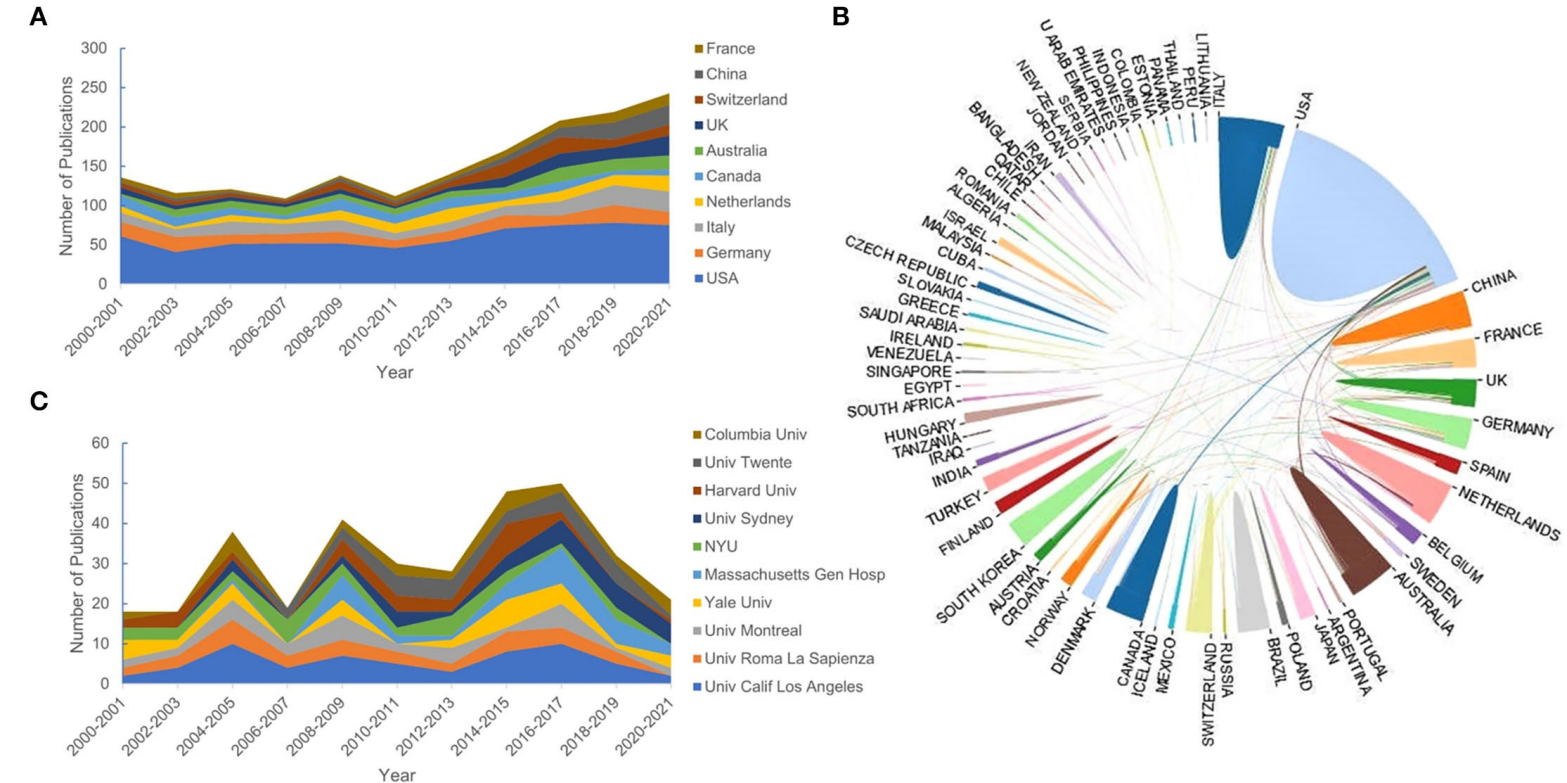
The top 10 countries and institutions. **(A)** The number of publications of the top 10 countries. **(B)** The cooperative relationship between countries. **(C)** The number of publications of the top 10 institutions.

Publications of QEEG research were obtained from 71 countries. About 89.92% of the publications were from the top 10 countries (Table 1). The trends of annual output from the top 10 countries are shown in Figure 3A. Most publications were from the United States (*n* = 657, 34.5%). The centrality analysis in CiteSpaceV represented the influence of a node, and a node was of great significance when the centrality value is greater than 0.1. Countries with a centrality value greater than 0.1 were the United States (*n* = 0.95) and Germany (*n* = 0.14), suggesting that publications from these two countries had a greater influence on QEEG research. The international cooperation between countries is shown in Figure 3C. The most frequent collaboration was observed between the United States and Canada, followed by Australia.

**Table 1:** Top 10 countries and institutions with the highest number of publications from 2000 to 2021.

**Rank**	**Country**	**Number of Publications**	**Centrality**	**Institution**	**Number of publications**	**Centrality**
1	USA	657	0.95	Univ Calif Los Angeles (USA)	60	0.26
2	Germany	168	0.14	Sapienza University of Rome (Italy)	35	0.11
3	Italy	167	0.06	Univ Montreal (Canada)	34	0.09
4	Netherlands	118	0.02	Yale University (USA)	33	0.09
5	Canada	115	0.07	Massachusetts Gen Hospital (USA)	32	0.06
6	Australia	112	0.01	NYU(USA)	31	0.08
7	UK	107	0.09	University Sydney (Australia)	31	0.05
8	Switzerland	105	0.03	Harvard University (USA)	30	0.06
9	China	85	0.02	University Twente(Netherlands)	30	0.04
10	France	78	0.01	Columbia University (USA)	27	0.01

A total of 2,433 institutions published QEEG-related articles. The trends of annual output from the top 10 institutions are shown in Figure 3B. The distribution of institutions is scattered. Table 1 lists the top 10 productive institutions, and 343 papers have been published by these institutions, accounting for 18.01% of the total publications. About 60% of the top 10 institutions are from the United States, including the University of California, Los Angeles, Yale University, Massachusetts General Hospital, New York University, Harvard University, and Columbia University.

### Analysis of Journals and Co-cited Journals

**Figure 4:**
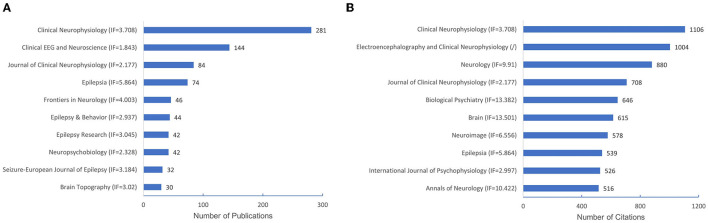
The number of publications of the top 10 journals and co-cited journals. **(A)** The number of publications of the top 10 journals. **(B)** The number of citations of the top 10 co-cited journals.

A total of 245 journals were involved, and the top 10 most active QEEG-related journals published 47.53% of the total publications (*n* = 905; Figure 4A). Among the top 10 journals in QEEG research, Clinical Neurophysiology (*n* = 281) was the most active journal, followed by Clinical EEG and Neuroscience (*n* = 144) and Journal of Clinical Neurophysiology (*n* = 84).

Co-cited journals were journals cited together by researchers, which usually reflected the foundation of a research field and were one of the most important indicators in bibliometric analysis. Half of the top 10 co-cited journals in QEEG research in neuropsychiatric disorders were published in Q1 according to JCR. Biological Psychiatry showed the highest impact factor (IF = 13.382). Clinical Neurophysiology (*n* = 1,106) ranked first in co-cited journals, followed by Electroencephalography and Clinical Neurophysiology (*n* = 1,004) and Neurology (*n* = 880), suggesting these journals were well recognized in QEEG research in the neuropsychiatric field (Figure 4B).

### Analysis of Authors

A total of 8,003 authors contributed to QEEG research in neuropsychiatric disorders. As presented in Table 2, six authors published over 25 articles. The most productive authors were Cook IA and Leuchter AF who published 36 articles in this field. Their research direction is mainly focused on the abnormality of QEEG in major depression and its rehabilitation by pharmacological treatments.

**Table 2:** Top 10 productive authors from 2000 to 2021.

**Rank**	**Author**	**H-index**	**Country**	**Institution**	**Count**	**Citation count**	**Yearly average citation count**
1	Cook IA	44	USA	University of California Los Angeles	36	1,464	67
2	Leuchter AF	43	USA	University of California Los Angeles	36	1,461	66
3	van Putten MJAM	16	Netherlands	University of Twente	29	1,067	49
4	Cagy M	2	-	Universidade Federal do Rio de Janeiro	26	199	9
5	Ribeiro P	-	-	-	26	208	9
6	Babiloni C	10	Italy	Sapienza University of Rome	25	905	41
7	Hunter AM	19	Scotland	University of Stirling	21	513	23
8	Piedade R	20	Brazil	Universidade Federal do Rio de Janeiro	21	183	8
9	Rossini PM	49	Italy	IRCCS San Raffaele Roma Rome	20	935	43
10	Del Percio C	41	Italy	Sapienza University of Rome	17	507	23

### Research Hot Topics Based on Keyword Analysis

**Figure 5:**
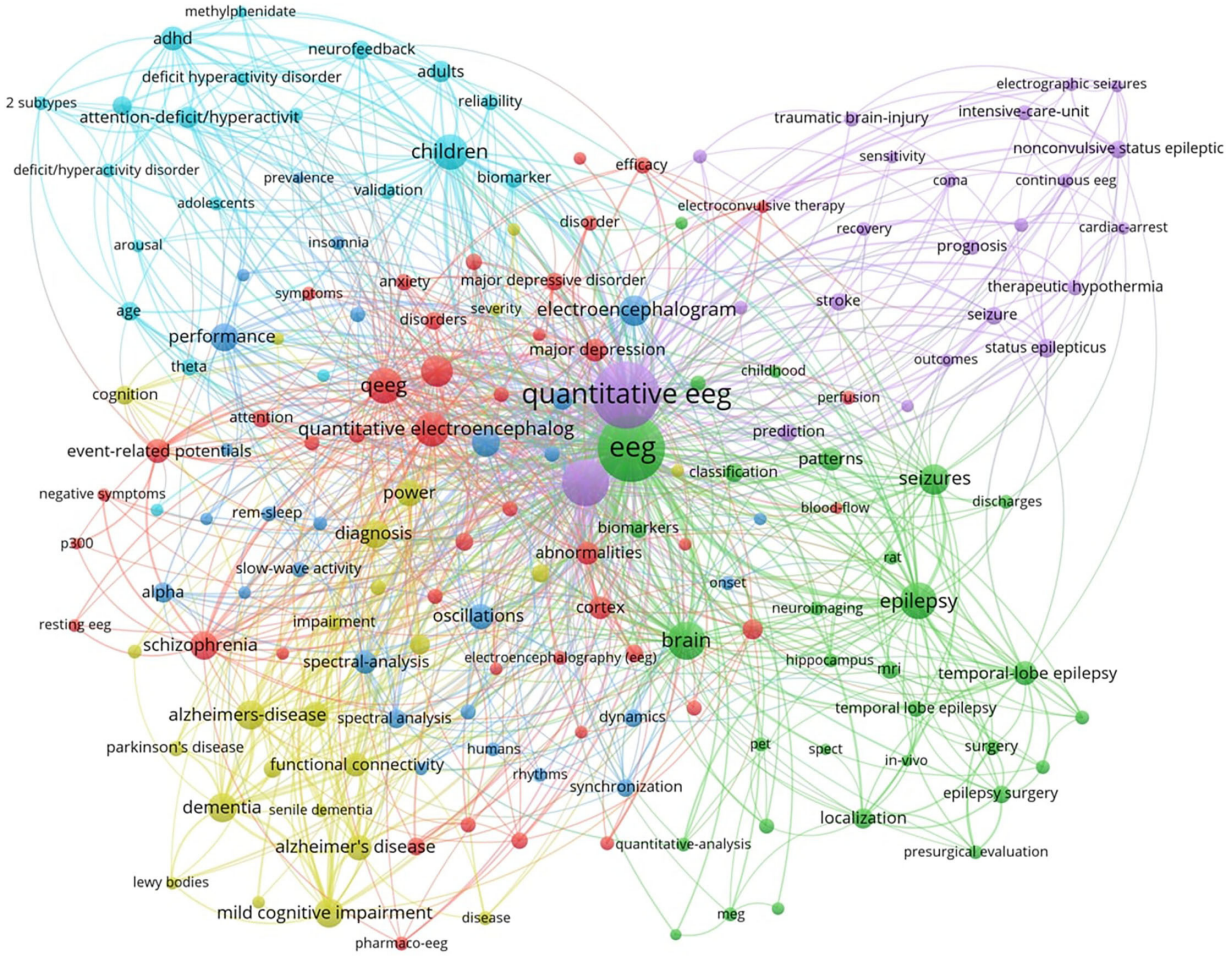
The keyword co-occurrence map.

**Figure 6:**
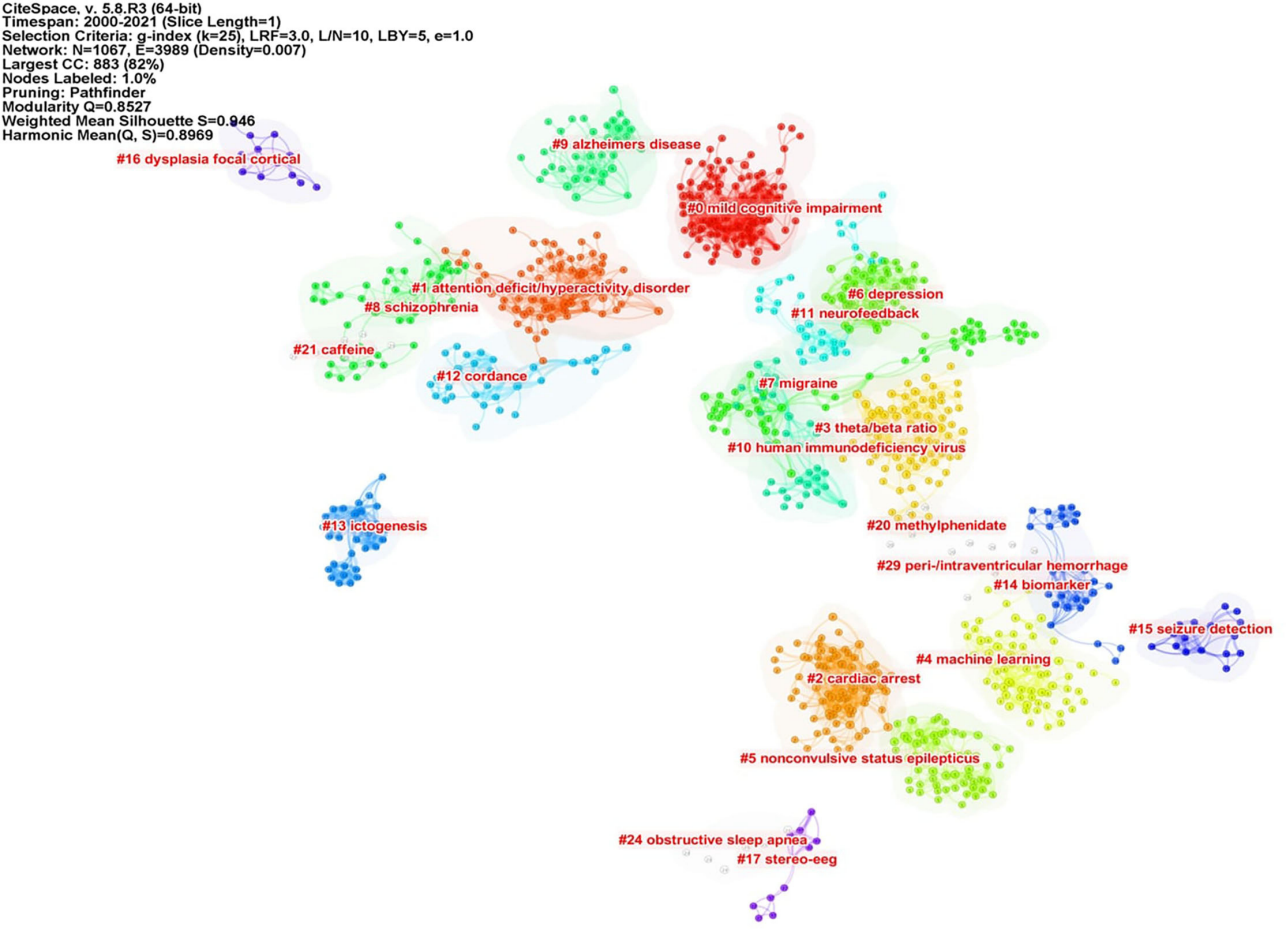
Cluster analysis of co-cited references.

To reveal the hot topics in the research field, we used VOSviewer to produce a keyword co-occurrence map (see Figure 6). The keyword co-occurrence map retrieved five major keyword clusters of QEEG research in neuropsychiatric disorders. As shown in Figure 5, each keyword cluster is depicted in a distinct color. That is, the blue cluster represents research in attention-deficit hyperactivity disorder (ADHD), the yellow cluster represents research in neurodegenerative disorders, such as Alzheimer's disease, the green cluster represents research in epilepsy, the purple cluster represents research in traumatic brain injury and related cerebrovascular diseases, and the red cluster represents research in psychiatric disorders, such as major depressive disorder and schizophrenia. These five research areas are currently hot topics in the research field.

### Knowledge Base by Cluster Analysis of Co-cited References

Co-cited references are co-cited articles in the reference lists of other articles. The co-citation network by CiteSpaceV revealed 1,067 nodes, 3,989 co-citation links, and 22 clusters. These clusters represented the knowledge base and networks of QEEG studies in neuropsychiatric disorders. Figure 6 shows all automatically extracted clusters. Each cluster is depicted with a unique color. The nodes in each cluster represent the co-cited documents, and the lines between the nodes represent the co-cited relationship. The labels of the clusters were extracted from the keywords of the citing publications, based on the latent semantic indexing (LSI) method. The general information about the co-citation clusters is summarized in Table 3, including the number of cited references, the average publication year of the cited references, and the silhouette value of each cluster. The silhouette value of a cluster ranges from 0 to 1, and a larger value indicated greater discrimination from other clusters (18). In addition, Table 4 presented the research disease categories and domains retrieved from the co-cited reference clusters. Supplementary Table S1 summarized highly co-cited references and the most relevant citing articles in each co-citation cluster.

**Table 3:** Basic information of the co-cited reference clusters.

**ID**	**Cluster name**	**Size**	**Avg (YR)**	**Silhouette**
0	Mild cognitive impairment (mci)	108	2005	0.90
1	Attention deficit/hyperactivity disorder	79	2000	0.93
2	Cardiac arrest	78	2012	0.94
3	Theta/beta ratio	78	2011	0.92
4	Machine learning	65	2017	0.94
5	Nonconvulsive status epilepticus	57	2016	0.90
6	Depression	52	2008	0.97
7	Migraine	50	2006	1.00
8	schizophrenia	46	1997	0.93
9	Alzheimers disease	39	2000	0.98
10	Human immunodeficiency virus (hiv)	36	2011	0.98
11	Neurofeedback	34	2008	0.95
12	Cordance	32	1999	0.95
13	Seizure anticipation	32	1998	0.99
14	Biomarker	29	2015	0.99
15	Seizure detection	16	2015	1.00
16	Dysplasia focal cortical	12	1996	1.00
17	Stereo-eeg	11	2014	1.00
20	Methylphenidate	9	2014	0.99
21	Caffeine	8	1996	1.00
24	Obstructive sleep apnea	7	2017	1.00
29	Peri-/intraventricular hemorrhage (pivh)	5	2008	0.99

*Size, number of publications in the cluster; Avg (YR), the average publication year of the references in the cluster*.

**Table 4:** Research disease categories and domains retrieved from the co-cited reference clusters.

**General disease category**	**Disease**	**Pathology**	**Diagnosis**	**Treatment**
Cerebrovascular diseases	Ischemic stroke		Cluster 2 cardiac arrest Cluster 7 migraine	Cluster2 cardiac arrest Cluster7 migraine
	Intracerebral hemorrhage		Cluster 29 peri-/intraventricular hemorrhage (pivh)	
Neurodegenerative diseases	Epilepsy	Cluster 17 stereo-eeg Cluster 16 dysplasia focal cortical Cluster 13 seizure anticipation	Cluster 5 nonconvulsive status epilepticus Cluster 15 seizure detection	Cluster 16 dysplasia focal cortical Cluster 15 seizure detection
	Alzheimer's disease	Cluster 8 schizophrenia Cluster 10 human immunodeficiency virus (hiv)	Cluster 0 mild cognitive impairment (mci) Cluster 9 alzheimer's disease	
	Parkinson's disease		Cluster 4 machine learning	Cluster 4 machine learning
Mental disorders	Attention-deficit/hyperactivity disorder	Cluster 1 attention deficit/hyperactivity disorder	Cluster 1 attention deficit/hyperactivity disorder Cluster 3theta/beta ratio	Cluster 11 neurofeedback Cluster 20 methylphenidate
	Depression			Cluster 6 depression Cluster 12 cordance Cluster 14 biomarker
Other	Substance abuse	Cluster 21 caffeine		
	Obstructive sleep apnea	Cluster 24 obstructive sleep apnea		

### Research Emerging Trends Based on Keyword Analysis

**Figure 7:**
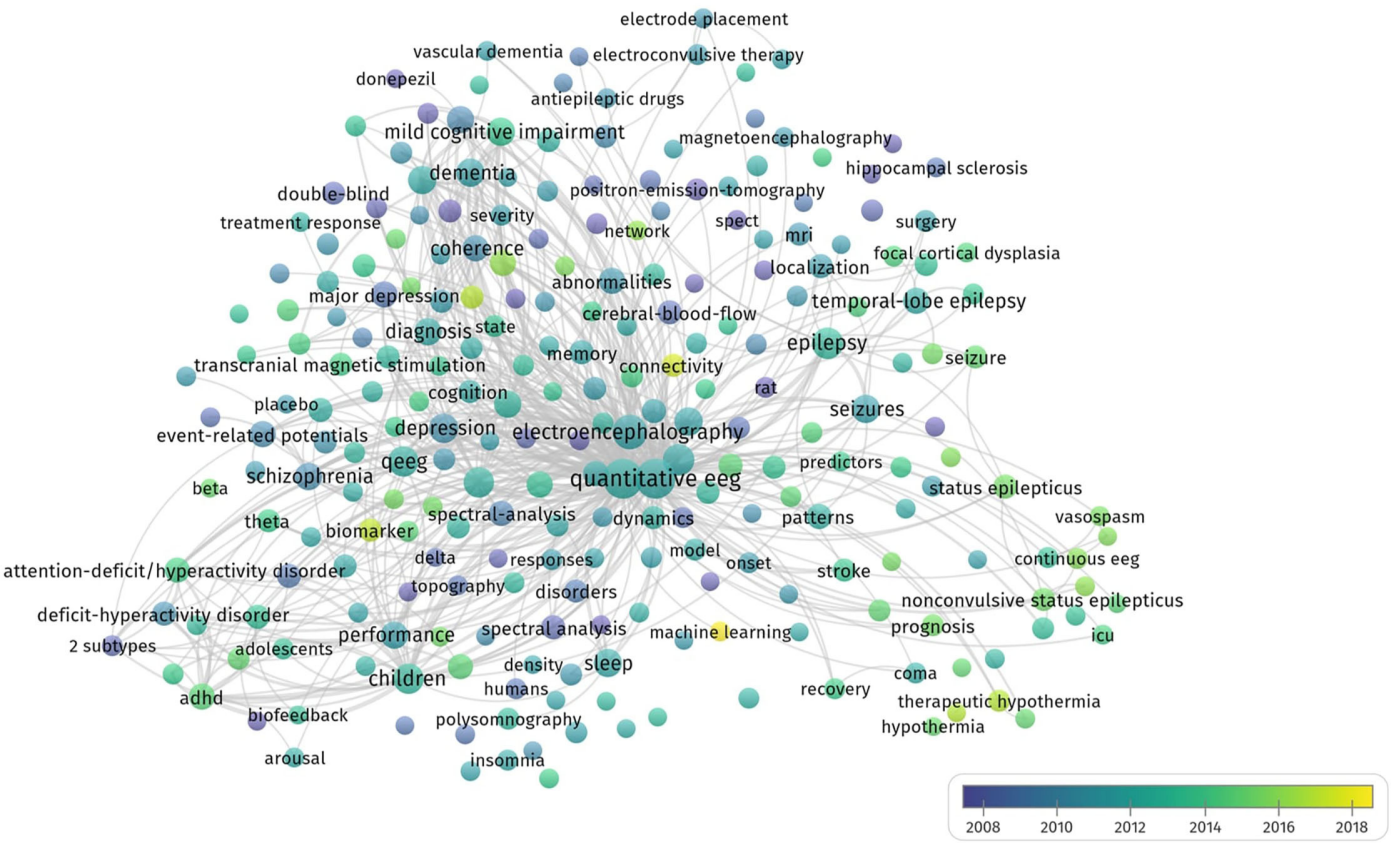
The overlay map.

We also used VOSviewer to produce the overlay map to show the latest emerging topics (Figure 7). In VOSviewer, we set the threshold of occurrence frequency to 15, and 253 of the total 7,458 keywords met the criteria. Among these keywords, “biomarker,” “connectivity,” and “machine learning” have emerged since 2018 and represented the future directions in QEEG studies in neuropsychiatric disorders. These emerging topics are depicted in yellow color in Figure 7.

## Discussion

### General Information

The current study applied a visualized bibliometric method to analyze the research hot spots, the knowledge base, and the emerging topics of the publications about QEEG in the neuropsychiatric research field. A total of 1,904 papers were collected based on 2000–2021 data from WoSCC. All papers were published by 2,433 institutions from 71 countries in 245 peer-reviewed journals with 57,237 co-cited references. The annual publication output and annual citation number revealed a steady growth in the research field. About 34.5% of the total publications were from the United States. Close cooperation between the United States, Canada, and Australia was found, suggesting their significant contribution to QEEG research in neuropsychiatric disorders. Among the 10 top institutions, 60% were from the United States, such as the University of California, Los Angeles, Yale University, Massachusetts General Hospital, New York University, Harvard University, and Columbia University. Among the top 10 authors, Cook IA and Leuchter AF published more studies and received higher co-citations, suggesting that their teams could be potential collaborators for researchers. Additionally, we found journals with high impact factors in our top 10 co-cited journals, such as Biological Psychiatry (IF = 13.382), Brain (IF = 13.501), and Annual of Neurology (IF = 10.422), which could be important sources of references.

### Hot Topics of QEEG Research in the Neuropsychiatric Disorders

According to the keyword co-occurrence map by VOSviewer, five clusters were retrieved and represented five major categories of neuropsychiatric diseases in QEEG research. The blue cluster represented the psychiatric disorder “ADHD.” ADHD is characterized by excessive restlessness and an extremely poor concentration span, resulting in impulsive and disruptive behavior. Bresnahan and Barry (19) suggested that QEEG might be used to differentiate ADHD adults from normal adults and adults who display the symptoms of ADHD without meeting the diagnostic criteria of ADHD. Elevated resting theta power and reduced alpha and beta power, together with elevated theta/alpha and theta/beta ratios, were found to be most reliably associated with ADHD (20, 21). Recently, novel measurements have emerged. For example, gamma power abnormalities might provide an opportunity to investigate the neurobiological mechanisms that underlie the clinical symptoms of ADHD (22–24).

The yellow cluster represented the neurodegeneration disorders, particularly Alzheimer's disease (AD) and Parkinson's disease (PD). Excessive slow wave activity has been shown in dementia of the Alzheimer's type that increases with disease progression (25). Compared to AD, the inter-hemispheric coherence values for the delta and theta bands in the fronto-temporo-central regions were higher in dementia with Lewy bodies (DLB). For patients with AD, the beta band was lower than DLB in almost all temporo-centro-parieto-occipital regions (26). Additionally, in patients with PD, abnormalities in QEEG, such as an increase in posterior theta power, were found with the occurrence of mild cognitive impairment or dementia (27). QEEG could also provide reliable biomarkers for objective monitoring of disease severity and progression in PD, as well as for promoting early diagnosis of nonmotor symptoms (28). For example, decreased dominant frequency and increased theta power, which reflect EEG slowing, were biomarkers of cognitive deterioration.

The green cluster represented “epilepsy” and “seizure,” which included the temporal lobe epilepsy, electroconvulsive-induced epilepsy, and so on. Larsson and colleagues showed that peak alpha frequency (PAF) variability was compromised in patients with epilepsy (29). Park et al. suggested that the automatic quantitative ictal high-gamma oscillation analysis may be effective in delineating the epileptogenic zone (30). QEEG background activity may also provide useful information on seizure duration. A higher theta power ratio in the temporal region contralateral to the epileptic focus may suggest a longer epilepsy duration (31).

The purple cluster represented traumatic brain injury (TBI) and related cerebrovascular diseases, such as stroke and subarachnoid hemorrhage (SAH). QEEG might predict the prognosis after TBI. In particular, measures of alpha power and variability were indicative of relatively better functional outcomes within the first year after TBI. This was hypothesized to reflect intact thalamo-cortical loops and thus the potential for recovery of consciousness even in the apparent absence of current consciousness (32). QEEG can also be used to identify patients at risk of cerebral infarction. In patients with SAH, there was a moderate correlation between transcranial Doppler/color-coded duplex sonography (TCD/TCCS) frequencies and QEEG alpha power reduction, but only QEEG could differentiate patients with and without cerebral infarction (33). Moreover, worsening alpha/delta ratio (ADR) on QEEG was a reliable predictor of delayed cerebral ischemia (DCI) in patients with aneurysmal SAH. Further studies are still needed to confirm the role of QEEG in the prediction of DCI (34).

The red cluster consisted of QEEG studies in several common psychiatric disorders, including major depressive disorder (MDD), anxiety, and schizophrenia (35, 36). Delta power values could be potentially used in the differential diagnosis between schizophrenia and depression. In patients with MDD, delta power over Fp1, Fp2, F4, and F8 regions was lower in comparison to schizophrenia patients (37). Impaired development of a resting-state brain network in adolescents with MDD may represent an intermediate phenotype that can be assessed with QEEG. Youth with MDD showed decreased resting connectivity in the alpha and theta frequency bands, particularly in the frontal cortex (38). In addition, Moon et. al found increased overall absolute delta power and relative gamma power as potential markers that could differentiate post-traumatic stress disorder (PTSD) from anxiety disorders (39).

### Knowledge Base of QEEG Research in the Neuropsychiatric Field

To better clarify the knowledge base of QEEG research in the neuropsychiatric field, we analyzed the cited literature and the citing literature in each co-citation cluster (Figure 6 and Table 3). A total of 22 clusters were extracted by CiteSpace through co-cited references analysis. These co-citation reference clusters could be classified into three disease categories: neurodegenerative diseases, cerebrovascular diseases, and mental disorders (Table 4). In these three disease categories, QEEG has been applied to investigate the pathological mechanisms, assist clinical diagnosis, and promote the selection of appropriate treatments. The most relevant co-cited articles and citing articles are listed in Supplementary Table S1.

In chronic neurodegenerative diseases, the majority of QEEG research is about Alzheimer's disease (AD) and Parkinson's disease (PD). In AD research, QEEG was mainly used to explore the pathology and diagnosis of AD. In the study of pathological mechanisms underlying AD, cluster 8 “schizophrenia” showed that there were similar QEEG characteristics between AD and schizophrenia (40–42) and that schizophrenia was associated with an elevated risk of developing AD (43), which suggested that the pathological mechanisms of these two diseases may be related. The co-cited references of cluster 10 “human immunodeficiency virus (HIV)” indicated that cortical source mapping by low-resolution brain electromagnetic source tomography (LORETA) of resting state EEG rhythms could characterize neurodegenerative disorders-induced cognitive impairment, such as Parkinson's disease related dementia (PDD) and Alzheimer's disease (AD) (44, 45), while the citing documents in this cluster showed that HIV research also applied LORETA in evaluating the cognitive functions in patients with HIV (46, 47). In the study of AD diagnosis, cluster 9 “Alzheimer's disease” indicated that QEEG can accurately differentiate the stage of AD (48–51). Cluster 0 “mild cognitive impairment (MCI)” suggested that QEEG was a valuable tool for the early diagnosis of AD (52–56). For PD patients (co-cited references of cluster 4 “machine learning” indicated that QEEG could provide reliable biomarkers for nonmotor symptom severity and progression (28, 57). Besides, the citing articles in this cluster pointed out that preoperative QEEG biomarkers could predict cognitive deterioration of PD after subthalamic deep brain stimulation with high accuracy by using a machine learning pipeline (58, 59).

In the study of acute neurodegenerative diseases, QEEG was also an important method to study epilepsy pathology, epilepsy prediction, epilepsy detection, and epilepsy treatment. In the research of pathological mechanisms of epilepsy, co-cited references of cluster 17 “stereo eeg” demonstrated that QEEG could be used to explore the desynchronization and synchronous discharge of neurons in different stages of epilepsy (60, 61). The citing literature of this cluster showed that quantitative stereo EEG could be used to analyze the inhibitory and promoting factors of seizures in inter-ictal period (62, 63). Based on the hypersynchronization hypothesis of epileptic seizures, cluster 13 “seizure anticipation” found that the trend of abnormal synchronization of neurons can be detected by QEEG nonlinear analysis to predict epileptic seizures (64–66). In terms of epilepsy detection, the co-cited literature of cluster 5 “nonconvulsive status epilepticus” showed that the use of QEEG could accurately diagnose epilepsy (67–69), and the citing literature of this cluster showed that QEEG could also be used to monitor nonconvulsive status epilepticus (70). In addition, cluster 15 “seizure detection” showed that QEEG combined with quantitative electromyography (EMG) can identify the characteristics of different epileptic subtypes (71, 72). In terms of epilepsy treatment, citing articles of cluster 16 “dysplasia focal cortical” indicated that the QEEG index can provide a reliable basis for determining epileptic focus before the surgical treatment of focal epilepsy (73), and the co-cited documents in this cluster proved that QEEG index could accurately predict the surgical prognosis of epilepsy (74, 75). Cluster 15 also showed that QEEG could help to predict and prevent sudden unexpected death in epilepsy (SUDEP) (76, 77).

In cerebrovascular diseases, according to the co-cited literature of cluster 2 “cardiac arrest” (CA) and cluster 7 “migraine,” EEG signals mainly come from the activities of pyramidal cells in the cerebral cortex, which are vulnerable to cerebral ischemia (78), so QEEG is suitable for detecting abnormal neural activities of ischemic stroke (IS) and evaluating the IS prognosis (79–81). The study of EEG characteristics of cerebral ischemia is also helpful to investigate other related diseases. Citing documents of cluster 2 suggested that cardiac arrest would cause secondary ischemic stroke. Therefore, even if cardiopulmonary resuscitation is successfully accepted, patients may have neurological sequelae. QEEG index can reflect brain activity in real time and assist doctors to judge the prognosis of patients with CA and take corresponding treatment in time (82, 83). The citing literature of cluster 7 showed that because migraine and ischemic stroke have similar EEG characteristics, it was speculated that the change in cerebral blood activity may be one of the manifestations of migraine (84, 85). In addition, according to cluster 29 “peri-/intraventricular hemorrhage (PIVH),” QEEG has also been used in the early diagnosis of intracerebral hemorrhage in premature infants in recent years (86–89). In brief, the knowledge base of QEEG in cerebrovascular diseases is mainly about monitoring and treating the abnormal brain functions related to cerebrovascular disease by using QEEG.

In the studies related to mental disorders, attention-deficit hyperactivity disorder (ADHD) and depression are two major application fields of QEEG. The cited and citing literature in cluster 1 “attention-deficit hyperactivity disorder” included various types of QEEG studies on ADHD, including the use of QEEG to study the etiology, diagnostic biomarkers, prognostic biomarkers, and add-on treatment of ADHD (20, 52, 86–93). First, the literature of cluster 1 indicated that QEEG could verify different etiological hypotheses of ADHD (90). Second, cluster 1 also denoted that QEEG could accurately judge the abnormal brain activities associated with ADHD (20, 93), and cluster 3 “theta/beta ratio” indicated that theta/beta ratio might be used as an index to identify ADHD subtypes (21, 94, 95). Third, the literature in cluster 20 “methylphenidate” mainly used the QEEG index to evaluate the efficacy of different drugs in the treatment of ADHD (96–98). Among them, methylphenidate has been proven to be a drug that can effectively alleviate the symptoms of ADHD (97, 98). Finally, it is particularly noteworthy that ADHD is the major application field of neurofeedback therapy. Cluster 11 “neurofeedback” showed that many studies have proved that neurofeedback therapy can effectively treat ADHD (54, 99–102), particularly when targeted, personalized neurofeedback treatment was applied (102). Moreover, the co-cited references of cluster 20 also showed the long-term efficacy of neurofeedback in the treatment of ADHD (103, 104). Therefore, the application of QEEG in the field of ADHD has a relatively good research foundation.

For the knowledge base of QEEG studies in other mental disorders, most studies on depression focused on evaluating the efficacy of antidepressants with QEEG indicators. The citing literature of cluster 6 “depression” (105, 106) and the co-cited literature in cluster 14 “biomarker” (107, 108) suggested that there may be methodological differences among studies and a lack of replications in this research area, so there is still no widely recognized QEEG index that can accurately predict the efficacy of antidepressants. Particularly, on the one hand, the co-cited literature in cluster 12 “cordance” suggested that cordance, a QEEG index that can comprehensively analyze relative EEG power and absolute EEG power and highlight the brain pathological activities (109), has not been able to predict the efficacy of antidepressants (110–112). On the other hand, the co-cited literature in cluster 6 (113–115) and the citing literature from cluster 12 (80, 116) showed that prefrontal theta cordance has the value of predicting the response of antidepressants, which indicated that the cordance index still has the potential for further research. Moreover, citing articles of cluster 14 argued that researchers can try using machine learning to explore QEEG biomarkers for evaluating the efficacy of antidepressants (117, 118).

Caffeine withdrawal response and sleep disorder are the remaining two clusters identified by CiteSpace, suggesting wide applications of QEEG in the neuropsychiatric field. The literature in cluster 21 “caffeine” showed that QEEG can be used to study the neural mechanism underlying the withdrawal response to drugs, such as caffeine and cocaine (119, 120). Cluster 24 “obstructive sleep apnea (OSA)” suggested that QEEG during sleep could help to reveal the pathological mechanism of OSA, while awake QEEG could evaluate the impact of OSA on cognitive functions (118, 121, 122).

### Emerging Trends and Future Direction of QEEG Research in Neuropsychiatric Disorders

Overlay visualization presented the time of emergence of the keywords and reflected the latest and emerging research topics. From the overlay map shown in Figure 7, we can see that the recently searched keywords are shown by yellow nodes. The emerging keywords were “biomarker,” “connectivity,” and “machine learning.”

As for QEEG biomarker research, recent studies started to cross-validate the prognostic value of previously suggested EEG biomarkers in larger independent datasets, since an increasing number of QEEG biomarkers in neuropsychiatric disorders were revealed in prior studies. For example, Ip and colleagues showed that alpha asymmetry seems to be the most promising EEG biomarker for the prediction of treatment response in women with MDD in comparison to alpha power, delta and theta activity at the anterior cingulate cortex (ACC) (123). Moreover, new QEEG biomarkers have also been investigated. Interictal high-frequency oscillation and modulation index have been found to improve the prediction accuracy of post-operative seizure outcomes (124).

QEEG-based functional connectivity has also been investigated in recent years as a diagnostic tool to predict the symptom severity of neuropsychiatric disorders. EEG functional connectivity has shown promising results as a diagnostic tool for AD. Similarly, in Down syndrome (DS) with Alzheimer's dementia, decreased alpha and increased delta coherence and weighted phase lag index were observed when compared to DS (125). EEG functional connectivity and complexity were used to predict depression severity among depressive patients. A significant negative relationship was found between graph metrics (i.e., degree and clustering coefficient) and depression severity in the alpha band, while the EEG complexity measures in alpha and delta bands by the nonlinear analysis were positively associated with symptom severity (126).

Another breakthrough is QEEG-based machine learning studies. Through machine learning, a compound of automatically extracted EEG biomarkers differentiated good vs. poor cognitive function of PD patients with higher accuracy than a single spectral EEG feature (58). More QEEG biomarkers (e.g., coherence, spectral, and event-related potentials) should be investigated and combined with machine learning or deep learning methods to predict the occurrence, severity, and treatment response for neuropsychiatric disorders.

### Strengths and Limitations

Our bibliometric study has several strengths. First of all, it is the first study to use the scientometric method to summarize the research history and development trends of QEEG studies in the neuropsychiatric field. It included the most comprehensive analysis, covering nearly all aspects of previous publications, and provided valuable information to QEEG researchers and helped them gain a better insight into the evolving research foci and trends. However, our study was also subjective to several limitations. First, the data come merely from WoSCC, and other databases, such as Embase or PubMed, were not searched, and hence this study may not completely represent all QEEG data. But notably, WoSCC is the most frequently used database for scientometric research. Second, the retrieved articles were restricted to those published in English, resulting in some linguistic bias.

## Conclusion

The present study performed a bibliometric analysis of the overall scientific output of QEEG research in the neuropsychiatric field from 2000 to 2021. During the last two decades, QEEG has been applied to reveal the pathological mechanisms, assist clinical diagnosis, and promote the selection of effective treatments for a variety of neuropsychiatric diseases, including neurodegenerative diseases, cerebrovascular diseases, and mental diseases. Studies in these disease categories and domains added to the knowledge base of this research field. The hot topics of research included five major neuropsychiatric disorders, including ADHD, neurodegenerative disorders like Alzheimer's and Parkinson's disease, traumatic brain injury and related cerebrovascular diseases, epilepsy and seizure, and other psychiatric diseases, such as MDD and schizophrenia. Besides, future studies should focus on cross-validating promising QEEG biomarkers, developing new biomarkers (e.g, functional connectivity and complexity), and extracting biomarkers by machine learning.

## Author Contributions

SY and JZ contributed equally to the analysis of the data and wrote the manuscript. SL collected the bibliometric data and prepared the figures. RZ interpreted the data and revised the manuscript. JZ revised the manuscript. XY and YW designed the study, interpreted the data, and revised the manuscript. All authors contributed to the article and approved the submitted version.

## Funding

This study was funded by the National Natural Science Foundation of China (grant nos: 31800928 and 72174082), Guangdong Provincial Philosophy and Social Sciences 13th Five-Year Plan Co-construction Project (grant no: GD18XXL04), and Guangzhou Philosophy and Social Sciences Development 13th Five-Year Plan Young Scholar Project of City of Rams (grant no: 2020GZQN42). Innovation and Entrepreneurship Training Program for College Students (project no: S202012121176).

## Conflict of Interest

The authors declare that the research was conducted in the absence of any commercial or financial relationships that could be construed as a potential conflict of interest.

## Publisher's Note

All claims expressed in this article are solely those of the authors and do not necessarily represent those of their affiliated organizations, or those of the publisher, the editors and the reviewers. Any product that may be evaluated in this article, or claim that may be made by its manufacturer, is not guaranteed or endorsed by the publisher.
