# Supplementary material for: Bibliometric Analysis of Quantitative Electroencephalogram Research in Neuropsychiatric Disorders From 2000 to 2021
Source: Front Psychiatry. 2022 May 23;13:830819. doi: 10.3389/fpsyt.2022.830819 (PMC9167960; doi:10.3389/fpsyt.2022.830819)
Supplement: Supplementary file 1 [file Table_1.DOCX]

**Table S1** **The summary of key citing and co-cited documents in each co-citation cluster**

| **Cluster** | **Document Type** | **Title** | **First Author** | **Publish Year** | **Source** |
| --- | --- | --- | --- | --- | --- |
| 0 | co-cited document | EEG dynamics in patients with Alzheimer's disease | Jeong JS | 2004 | CLIN NEUROPHYSIOL |
| 0 | co-cited document | Sources of cortical rhythms change as a function of cognitive impairment in pathological aging: a multicenter study | Babiloni C | 2006 | CLIN NEUROPHYSIOL |
| 0 | co-cited document | Mapping distributed sources of cortical rhythms in mild Alzheimer's disease. A multicentric EEG study | Babiloni C | 2004 | NEUROIMAGE |
| 0 | citing document | Evidence-Based Evaluation of Diagnostic Accuracy of Resting EEG in Dementia and Mild Cognitive Impairment | Jelic V | 2009 | CLINICAL EEG AND NEUROSCIENCE |
| 0 | citing document | Electroencephalography and event-related potentials as biomarkers of mild cognitive impairment and mild Alzheimer's disease | Jackson CE | 2008 | ALZHEIMERS & DEMENTIA |
| 0 | citing document | Quantitative electroencephalography power and coherence measurements in the diagnosis of mild and moderate Alzheimer's disease | Fonseca LC | 2011 | ARQUIVOS DE NEURO-PSIQUIATRIA |
| 1 | co-cited document | Electroencephalogram differences in two subtypes of attention-deficit/hyperactivity disorder | Clarke AR | 2001 | PSYCHOPHYSIOLOGY |
| 1 | co-cited document | Conventional and quantitative electroencephalography in psychiatry | Hughes JR | 1999 | J NEUROPSYCH CLIN N |
| 1 | co-cited document | A review of electrophysiology in attention-deficit/hyperactivity disorder: I. Qualitative and quantitative electroencephalography | Barry RJ | 2003 | CLIN NEUROPHYSIOL |
| 1 | co-cited document | Assessing attention deficit hyperactivity disorder via quantitative electroencephalography: an initial validation study | Monastra VJ | 2001 | NEUROPSYCHOLOGY |
| 1 | citing document | EEG coherence in attention-deficit/hyperactivity disorder: a comparative study of two DSM-IV types | Barry RJEEG | 2002 | CLINICAL NEUROPHYSIOLOGY |
| 1 | citing document | Resting EEG theta activity predicts cognitive performance in attention-deficit hyperactivity disorder | Hermens DFR | 2005 | PEDIATRIC NEUROLOGY |
| 1 | citing document | The clinical role of computerized EEG in the evaluation and treatment of learning and attention disorders in children and adolescents | Chabot RJT | 2001 | JOURNAL OF NEUROPSYCHIATRY AND CLINICAL NEUROSCIENCES |
| 1 | citing document | EEG correlates of methylphenidate response in ADHD: Association with cognitive and behavioral measures | Loo SKEEG | 2004 | JOURNAL OF CLINICAL NEUROPHYSIOLOGY |
| 2 | co-cited document | EEG in ischaemic stroke: quantitative EEG can uniquely inform (sub-)acute prognoses and clinical management | Finnigan S | 2013 | CLIN NEUROPHYSIOL |
| 2 | co-cited document | Recommendations on the use of EEG monitoring in critically ill patients: consensus statement from the neurointensive care section of the ESICM | Claassen J | 2013 | INTENS CARE MED |
| 2 | citing document | Prognostic value of electroencephalography (EEG) for brain injury after cardiopulmonary resuscitation | Feng GP | 2016 | NEUROLOGICAL SCIENCES |
| 2 | citing document | Consensus Statement on Continuous EEG in Critically Ill Adults and Children, Part I: Indications, JOURNAL OF CLINICAL NEUROPHYSIOLOGY | Herman ST | 2015 | JOURNAL OF CLINICAL NEUROPHYSIOLOGY |
| 3 | co-cited document | A decade of EEG Theta/Beta Ratio Research in ADHD: a meta-analysis | Arns M | 2013 | J ATTEN DISORD |
| 3 | co-cited document | The quantitative EEG theta/beta ratio in attention deficit/hyperactivity disorder and normal controls: sensitivity, specificity, and behavioral correlates | Ogrim G | 2012 | PSYCHIAT RES |
| 3 | citing document | A Decade of EEG Theta/Beta Ratio Research in ADHD: A Meta-Analysis | Arns MA | 2013 | JOURNAL OF ATTENTION DISORDERS |
| 3 | citing document | Characterization of the Theta to Beta Ratio in ADHD: Identifying Potential Sources of Heterogeneity | Loo SK | 2013 | JOURNAL OF ATTENTION DISORDERS |
| 4 | co-cited document | Clinical correlates of quantitative EEG in Parkinson disease: A systematic review | Geraedts VJ | 2018 | NEUROLOGY |
| 4 | co-cited document | Quantitative EEG Applying the Statistical Recognition Pattern Method: A Useful Tool in Dementia Diagnostic Workup | Engedal K | 2015 | DEMENT GERIATR COGN |
| 4 | citing document | Preoperative Electroencephalography-Based Machine Learning Predicts Cognitive Deterioration after Subthalamic Deep Brain Stimulation | Geraedts VJ | 2021 | MOVEMENT DISORDERS |
| 4 | citing document | Machine learning for automated EEG-based biomarkers of cognitive impairment during Deep Brain Stimulation screening in patients with Parkinson’s Disease | Geraedts VJ | 2021 | CLINICAL NEUROPHYSIOLOGY |
| 5 | co-cited document | Sensitivity of quantitative EEG for seizure identification in the intensive care unit | Haider HA | 2016 | NEUROLOGY |
| 5 | co-cited document | Diagnostic Accuracy of Electrographic Seizure Detection by Neurophysiologists and Non-Neurophysiologists in the Adult ICU Using a Panel of Quantitative EEG Trends | Swisher CB | 2015 | J CLIN NEUROPHYSIOL |
| 5 | co-cited document | Defining abnormal slow EEG activity in acute ischaemic stroke: Delta/alpha ratio as an optimal QEEG index | Finnigan S | 2016 | CLIN NEUROPHYSIOL |
| 5 | citing document | Color density spectral array of bilateral bispectral index system: Electroencephalographic correlate in comatose patients with nonconvulsive status epilepticus | Hernandez-Hernandez HM | 2016 | SEIZURE-EUROPEAN JOURNAL OF EPILEPSY |
| 6 | citing document | Use of Clinical Neurophysiology for the Selection of Medication in the Treatment of Major Depressive Disorder: the State of the Evidence | Leuchter AF | 2009 | CLINICAL EEG AND NEUROSCIENCE |
| 6 | citing document | Biomarkers to Predict Antidepressant Response | Leuchter AF | 2010 | CURRENT PSYCHIATRY REPORTS |
| 6 | co-cited document | Early reduction in prefrontal theta QEEG cordance value predicts response to venlafaxine treatment in patients with resistant depressive disorder | Bares M | 2008 | EUR PSYCHIAT |
| 6 | co-cited document | Changes in QEEG prefrontal cordance as a predictor of response to antidepressants in patients with treatment resistant depressive disorder: a pilot study | Bares M | 2007 | J PSYCHIATR RES |
| 6 | co-cited document | Changes in prefrontal activity characterize clinical response in SSRI nonresponders: a pilot study | Cook IA | 2005 | J PSYCHIATR RES |
| 7 | co-cited document | Quantitative EEG indices of sub-acute ischaemic stroke correlate with clinical outcomes | Finnigan SP | 2007 | CLIN NEUROPHYSIOL |
| 7 | co-cited document | The revised brain symmetry index | van Putten MJAM | 2007 | CLIN NEUROPHYSIOL |
| 7 | citing document | The occipital alpha rhythm related to the "migraine cycle" and headache burden: A blinded, controlled longitudinal study | Bjork MH | 2009 | CLINICAL NEUROPHYSIOLOGY |
| 7 | citing document | Interictal quantitative EEG in migraine: a blinded controlled study | Bjork MH | 2009 | JOURNAL OF HEADACHE AND PAIN |
| 8 | co-cited document | Correlations of topographical EEG features with clinical severity in mild and moderate dementia of Alzheimer type | Chiaramonti R | 1997 | NEUROPSYCHOBIOLOGY |
| 8 | co-cited document | Low resolution brain electromagnetic tomography (LORETA) functional imaging in acute, neuroleptic-naive, first-episode, productive schizophrenia | Pascual-Marqui RD | 1999 | PSYCHIAT RES-NEUROIM |
| 8 | citing document | Source distribution of neuromagnetic slow wave activity in schizophrenic and depressive patients | Wienbruch C | 2003 | CLINICAL NEUROPHYSIOLOGY |
| 9 | co-cited document | Quantitative electroencephalography in mild cognitive impairment: longitudinal changes and possible prediction of Alzheimer's disease | Jelic V | 2000 | NEUROBIOL AGING |
| 9 | co-cited document | Discrimination of Alzheimer's disease and mild cognitive impairment by equivalent EEG sources: a cross-sectional and longitudinal study | Huang C | 2000 | CLIN NEUROPHYSIOL |
| 9 | co-cited document | EEG spectral profile to stage Alzheimer's disease. | Rodriguez G | 1999 | CLIN NEUROPHYSIOL |
| 9 | citing document | A critical discussion of the role of neuroimaging in mild cognitive impairment | Wolf H | 2003 | ACTA NEUROLOGICA SCANDINAVICA |
| 10 | co-cited document | Longitudinal EEG changes correlate with cognitive measure deterioration in Parkinson's disease | Caviness JN | 2015 | J PARKINSON DIS |
| 10 | co-cited document | Cortical sources of resting state electroencephalographic rhythms in Parkinson's disease related dementia and Alzheimer's disease | Babiloni C | 2011 | CLIN NEUROPHYSIOL |
| 10 | citing document | Abnormal cortical sources of resting state electroencephalographic rhythms in single treatment-naive HIV individuals: A statistical z-score index | Babiloni C | 2016 | CLIN NEUROPHYSIOL |
| 10 | citing document | Cortical sources of resting-state EEG rhythms in "experienced" HIV subjects under antiretroviral therapy | Babiloni C | 2014 | CLIN NEUROPHYSIOL |
| 11 | co-cited document | Efficacy of neurofeedback treatment in ADHD: the effects on inattention, impulsivity and hyperactivity: a meta-analysis | Arns M | 2009 | CLIN EEG NEUROSCI |
| 11 | co-cited document | The effects of QEEG-informed neurofeedback in ADHD: an open-label pilot study | Arns M | 2012 | APPL PSYCHOPHYS BIOF |
| 11 | co-cited document | Is neurofeedback an efficacious treatment for ADHD? A randomised controlled clinical trial | Gevensleben H | 2009 | J CHILD PSYCHOL PSYC |
| 11 | citing document | Effects of Neurofeedback Versus Stimulant Medication in Attention-Deficit/Hyperactivity Disorder: A Randomized Pilot Study | Ogrim GE | 2013 | JOURNAL OF CHILD AND ADOLESCENT PSYCHOPHARMACOLOGY |
| 11 | citing document | Quantitative EEG Neurofeedback for the Treatment of Pediatric Attention-Deficit/Hyperactivity Disorder, Autism Spectrum Disorders, Learning Disorders, and Epilepsy | Hurt E | 2014 | CHILD AND ADOLESCENT PSYCHIATRIC CLINICS OF NORTH AMERICA |
| 11 | citing document | Neurofeedback An Emerging Technology for Treating Central Nervous System Dysregulation | Larsen SN | 2013 | PSYCHIATRIC CLINICS OF NORTH AMERICA |
| 12 | co-cited document | Changes in brain function of depressed subjects during treatment with placebo | Leuchter AF | 2002 | AM J PSYCHIAT |
| 12 | co-cited document | Early Changes in Prefrontal Activity Characterize Clinical Responders to Antidepressants | Cook IA | 2002 | NEUROPSYCHOPHARMACOL |
| 12 | co-cited document | Neurophysiologic predictors of treatment response to fluoxetine in major depression | Cook IA | 1999 | PSYCHIAT RES |
| 12 | citing document | The promise of the quantitative electroencephalogram as a predictor of antidepressant treatment outcomes in major depressive disorder | Hunter AM | 2007 | PSYCHIATRIC CLINICS OF NORTH AMERICA |
| 12 | citing document | Pretreatment neurophysiological and clinical characteristics of placebo responders in treatment trials for major depression | Leuchter AFP | 2004 | PSYCHOPHARMACOLOGY |
| 13 | co-cited document | Epileptic seizures can be anticipated by non-linear analysis | Martinerie J | 1998 | NAT MED |
| 13 | co-cited document | Spatio-temporal characterizations of non-linear changes in intracranial activities prior to human temporal lobe seizures | Le Van Quyen M | 2000 | EUR J NEUROSCI |
| 13 | co-cited document | Can Epileptic Seizures be Predicted? Evidence from Nonlinear Time Series Analysis of Brain Electrical Activity | Lehnertz K | 1998 | PHYS REV LETT |
| 13 | citing document | Seizure anticipation in pediatric epilepsy: Use of Kolmogorov entropy | van Drongelen WS | 2003 | PEDIATRIC NEUROLOGY |
| 13 | citing document | Prediction of epileptic seizures | Litt BP | 2002 | LANCET NEUROLOGY |
| 14 | co-cited document | EEG alpha asymmetry as a gender-specific predictor of outcome to acute treatment with different antidepressant medications in the randomized iSPOT-D study | Arns M | 2016 | CLIN NEUROPHYSIOL |
| 14 | co-cited document | Electroencephalographic Biomarkers for Treatment Response Prediction in Major Depressive Illness: A Meta-Analysis | Widge AS | 2019 | AM J PSYCHIAT |
| 14 | citing document | Leveraging Machine Learning Approaches for Predicting Antidepressant Treatment Response Using Electroencephalography (EEG) and Clinical Data | Jaworska NL | 2019 | FRONTIERS IN PSYCHIATRY |
| 14 | citing document | Quantitative Electroencephalography in Guiding Treatment of Major Depression | Schiller MJ | 2019 | FRONTIERS IN PSYCHIATRY |
| 15 | co-cited document | Quantitative analysis of surface electromyography: Biomarkers for convulsive seizures | Beniczky S | 2016 | CLIN NEUROPHYSIOL |
| 15 | citing document | Automated real-time detection of tonic-clonic seizures using a wearable EMG device | Beniczky SA | 2018 | NEUROLOGY |
| 15 | co-cited document | Risk factors of postictal generalized EEG suppression in generalized convulsive seizures | Alexandre V | 2015 | NEUROLOGY |
| 15 | citing document | Ictal quantitative surface electromyography correlates with postictal EEG suppression | Arbune AA | 2020 | NEUROLOGY |
| 16 | citing document | Correlation between inter-ictal regional cerebral blood flow and sphenoidal electrodes - recorded inter-ictal spikes in mesial temporal lobe epilepsy | Lemesle MC | 2000 | NEUROLOGICAL RESEARCH |
| 16 | co-cited document | Single photon emission computed tomography-EEG relations in temporal lobe epilepsy | Lee BI | 1997 | NEUROLOGY |
| 16 | co-cited document | Routine EEG and temporal lobe epilepsy: relation to long-term EEG monitoring, quantitative MRI, and operative outcome | Cascino GD | 1996 | EPILEPSIA |
| 17 | co-cited document | Synchronization and desynchronization in epilepsy: controversies and hypotheses | Jiruska P | 2013 | J PHYSIOL-LONDON |
| 17 | co-cited document | Facilitation of epileptic activity during sleep is mediated by high amplitude slow waves | Frauscher B | 2015 | BRAIN |
| 17 | citing document | Suppression of interictal spikes during phasic rapid eye movement sleep: a quantitative stereo-electroencephalography study | Campana CS | 2017 | JOURNAL OF SLEEP RESEARCH |
| 17 | citing document | Correlating Interictal Spikes with Sigma and Delta Dynamics during Non-Rapid-Eye-Movement-Sleep | Zubler FC | 2017 | FRONTIERS IN NEUROLOGY |
| 20 | co-cited document | Predicting the clinical outcome of stimulant medication in pediatric attention-deficit/hyperactivity disorder: data from quantitative electroencephalography, event-related potentials, and a go/no-go test | Ogrim G | 2014 | NEUROPSYCH DIS TREAT |
| 20 | citing document | Personalized at-home neurofeedback compared with long-acting methylphenidate in an european non-inferiority randomized trial in children with ADHD | Bioulac S | 2019 | BMC PSYCHIATRY |
| 20 | citing document | A randomized controlled trial into the effects of neurofeedback, methylphenidate, and physical activity on EEG power spectra in children with ADHD | Janssen TW | 2016 | JOURNAL OF CHILD PSYCHOLOGY AND PSYCHIATRY |
| 20 | co-cited document | Evaluation of neurofeedback in ADHD: the long and winding road | Arns M | 2014 | BIOL PSYCHOL |
| 20 | co-cited document | Sustained effects of neurofeedback in ADHD: a systematic review and meta-analysis | Van Doren J | 2019 | EUR CHILD ADOLES PSY |
| 21 | co-cited document | Quantitative electroencephalographic analyses in cocaine-preferring polysubstance abusers during abstinence | ROEMER RA | 1995 | PSYCHIAT RES |
| 21 | citing document | Caffeine withdrawal increases cerebral blood flow velocity and alters quantitative electroencephalography (EEG) activity | Jones HEC | 2000 | PSYCHOPHARMACOLOGY |
| 24 | co-cited document | An automated algorithm to identify and reject artefacts for quantitative EEG analysis during sleep in patients with sleep-disordered breathing | DRozario AL | 2015 | SLEEP BREATH |
| 24 | co-cited document | Quantitative electroencephalogram measures in adult obstructive sleep apnea - Potential biomarkers of neurobehavioural functioning | DRozario AL | 2017 | SLEEP MED REV |
| 29 | co-cited document | Electroencephalography in premature and full-term infants. Developmental features and glossary | Andre M | 2010 | NEUROPHYSIOL CLIN |
| 29 | co-cited document | Normal EEG of premature infants born between 24 and 30 weeks gestational age: terminology, definitions and maturation aspects | Vecchierini MF | 2007 | NEUROPHYSIOL CLIN |
| 29 | co-cited document | Decreased aEEG continuity and baseline variability in the first 48 hours of life associated with poor short-term outcome in neonates born before 29 weeks gestation | Bowen JR | 2010 | PEDIATR RES |
| 29 | citing document | Early abnormal amplitude-integrated electroencephalography (aEEG) is associated with adverse short-term outcome in premature infants | Soubasi VE | 2012 | EUROPEAN JOURNAL OF PAEDIATRIC NEUROLOGY |
|  | citing document | Quantitative electroencephalography measures in rapid eye movement and nonrapid eye movement sleep are associated with apnea-hypopnea index and nocturnal hypoxemia in men | Appleton SL | 2019 | SLEEP |
|  | citing document | Sleep spindle activity correlates with implicit statistical learning consolidation in untreated obstructive sleep apnea patients | Stevens DS | 2021 | SLEEP MEDICINE |
